# The impact of energy‐based devices on sebum in acne vulgaris: A systematic review

**DOI:** 10.1111/jocd.16466

**Published:** 2024-08-11

**Authors:** Dana Jaalouk, Anika Pulumati, Yanci A. Algarin, Jasmine Humeda, David J. Goldberg

**Affiliations:** ^1^ Department of Dermatology and Cutaneous Surgery University of Miami Leonard M. Miller School of Medicine Miami Florida USA; ^2^ University of Missouri‐Kansas City School of Medicine Kansas City Missouri USA; ^3^ Eastern Virginia Medical School Norfolk Virginia USA; ^4^ Division of Dermatology University of Louisville Louisville Kentucky USA; ^5^ Skin Laser & Surgery Specialists, A Division of Schweiger Dermatology Group New York New York USA; ^6^ Icahn School of Medicine, Mount Sinai New York New York USA

**Keywords:** acne vulgaris, energy‐based devices, sebum

## Abstract

**Background:**

Acne vulgaris (AV) is a widespread inflammatory skin condition associated with increased sebum production, abnormal keratinization, bacterial overgrowth, and inflammation. Overactive sebaceous glands (SGs) produce excess sebum, promote *Cutibacterium acnes* growth, and affect acne development. Energy‐based treatments (EBDs), including light therapy, photodynamic therapy (PDT), lasers, and radiofrequency (RF) devices, have emerged as effective treatment options. As the use of EBDs becomes more widespread, it is imperative to understand their effects on skin parameters, such as sebum, in AV.

**Methods:**

Searches were conducted in Embase, PubMed, Web of Science, and the Cochrane Library. The studies included were randomized and nonrandomized trials on facial AV that used EBDs and featured objective casual sebum level (CSL) measurements via Sebumeter. Data synthesis involved percentage reductions in CSL at follow‐ups compared to baseline.

**Results:**

Twenty‐three studies were analyzed. PDT and RF consistently reduced CSL by 30%–40% and 30%–35%, respectively. Laser therapy showed lesser reductions, whereas light therapy varied significantly and studies had a high risk of bias. All EBD therapies were more effective than no treatment and PDT was superior to light monotherapy. Laser therapy combined with fractional microneedling radiofrequency (FMR) or as a standalone was more effective than laser alone.

**Conclusion:**

Noninvasive sebum measurement provides valuable insights into AV treatment efficacy. PDT, lasers, especially the 1450‐nm diode laser, and FMR are promising for reducing sebum. Standardization of measurement techniques and further research are vital for enhancing treatment personalization, reducing side effects, and improving AV management.

## 1 INTRODUCTION

Sebum, produced by sebaceous glands (SGs) in the dermis, is essential for skin health, maintaining hydration, and protecting against bacteria. However, its overproduction can lead to acne vulgaris (AV), a common skin condition characterized by comedones, papules, and pustules.^1^ AV's etiology includes increased sebum, abnormal keratinization, overgrowth of *Cutibacterium acnes* (*C. acnes*), and inflammation, with excess sebum being a major contributor to acne development.^1^, ^2^

AV management includes topical and oral medications and physical treatments such as light and lasers. Hormonal therapies and oral isotretinoin are the only treatments known to effectively reduce sebum production but are associated with significant risks, including feminization and teratogenicity, underscoring the need for safer sebum‐regulating treatments.^3^

The field of energy‐based devices (EBDs), such as lasers, light therapies, photodynamic therapy (PDT), and radiofrequency (RF) devices is rapidly evolving. EBDs treat AV by targeting *C. acnes* bacteria, reducing inflammation, and/or impacting SG activity with variable efficacy. An early approach to AV with EBDs included the use of natural ultraviolet (UV) light, a technique within PDT.^4^ Additionally, visible‐light lasers and intense pulsed light (IPL) devices have been employed to combat *C. acnes*, either as standalone treatments or in conjunction with PDT.^5^ Infrared lasers and RF technologies utilize heat to target water in the skin surrounding the SGs, causing thermal destruction of the glands. Histological examinations have documented shrinkage in SG size, similar to the effects observed with oral isotretinoin usage.^6^ A significant advancement is the FDA‐approved 1726‐nm laser, marketed under the brand names AviClear® and Accure®. This laser specifically targets and destroys SGs through selective photothermolysis, utilizing sebum as its primary chromophore. This innovation not only treats AV effectively by reducing sebum production but also results in fewer adverse events than earlier EBDs. Its development emphasizes the importance of SGs in AV pathology. As these EBDs become more prominent, understanding their impact on skin health and sebum production is crucial.

Noninvasive sebum measurements offer objective insights into AV treatment efficacy.^2^ Key metrics include casual sebum level (CSL) and sebum excretion rate (SER), with CSL often preferred for its simplicity and efficiency in clinical trials.^2^ However, research on the effects of EBDs on CSL in AV is limited, highlighting the need for comprehensive data analysis to gain insights.

This systematic review evaluates the effects of light, PDT, laser, and RF treatments on CSL in facial AV, comparing these therapies with other acne treatments and controls. It seeks to enhance the general knowledge of acne treatment outcomes, potentially guiding future therapeutic advancements and improving patient care.

## 2 METHODS

### 2.1 Eligibility criteria

Eligible studies included randomized and nonrandomized trials with at least two treatment arms, written in English, published in peer‐reviewed journals, and focused on facial AV. They must fit into one of the following therapy groups: light, PDT, laser, or RF. Objective CSL measurements in micrograms per square centimeter (μg/cm^2^) using a Sebumeter (Courage and Khazaka GmbH) were required. Sebumeter was selected because of its widespread use, reproducibility, and minimal uncertainty.^2^

We excluded single‐arm trials, case reports, gray literature, non‐English articles, studies on other conditions, and those measuring sebum in nonfacial areas. Additionally, studies not utilizing a Sebumeter or specifying the measurement instrument were excluded.

### 2.2 Search strategy

We search Embase (December 12, 2023), MEDLINE (January 15, 2024), Web of Science (December 13, 2023), and CENTRAL (November 29, 2023) using key phrases such as “acne vulgaris” AND “laser,” “light,” “photodynamic therapy,” OR “radiofrequency” AND “sebum.” See the Appendix S1 for detailed search strategy.

### 2.3 Selection process

Search results were imported into the Covidence systematic review platform for screening. Two authors independently reviewed titles, abstracts, and full texts, assigning inclusion or exclusion statuses. Discrepancies were resolved by a third reviewer.

### 2.4 Risk of bias assessment

The risk of bias assessment was conducted in Covidence using the revised Cochrane ‘Risk of Bias’ tool for randomized trials (RoB 2.0)^7^ and the Cochrane Risk Of Bias In Nonrandomized Studies‐of Interventions (ROBINS‐I) tool.^8^ The Risk‐of‐Bias VISualization (robvis) tool was used to create Traffic Light Plots and unweighted Summary Plots for visualization.^9^

### 2.5 Data collection

Two independent authors collected data via Covidence. Study specifics (authors, year, country), sample demographics, and participant details (acne severity, Fitzpatrick skin type) were recorded. Additionally, research design, intervention details (type, duration, dose/parameters, timing), and sebum measurement techniques (premeasurement protocol, location) were documented. Baseline and follow‐up sebum measurements were recorded, with end‐of‐treatment (EOT) measurements used if no follow‐up was provided. When data was only available in graph form, it was extracted using WebPlotDigitizer. If necessary, the corresponding authors were contacted for missing data or clarification.

### 2.6 Effect measure

The primary effect measure was the percentage reduction in CSLs from baseline at follow‐up or EOT if no follow‐up was performed.

### 2.7 Synthesis methods

We reported the percentage reduction in CSL from baseline. For studies without percentage data, we calculated it from baseline and follow‐up values. When multiple facial locations were measured, the facial average was used.

The studies were grouped into four main intervention categories: light therapy, PDT, laser therapy, and RF therapy. They were subdivided into 18 potential groups based on the study's comparison framework and organized alphabetically for tabulation.

## 3 RESULTS

### 3.1 Search strategy results

After searching four databases, 1600 studies were found, 1065 were screened, 66 were fully reviewed, and 23 were included (Figure 1). Four studies were organized into the Light intervention group, nine into PDT group, five into the Laser group, and five into the RF group. Table 1 reviews the treatments found and their mechanisms of sebum reduction.

**FIGURE 1:**
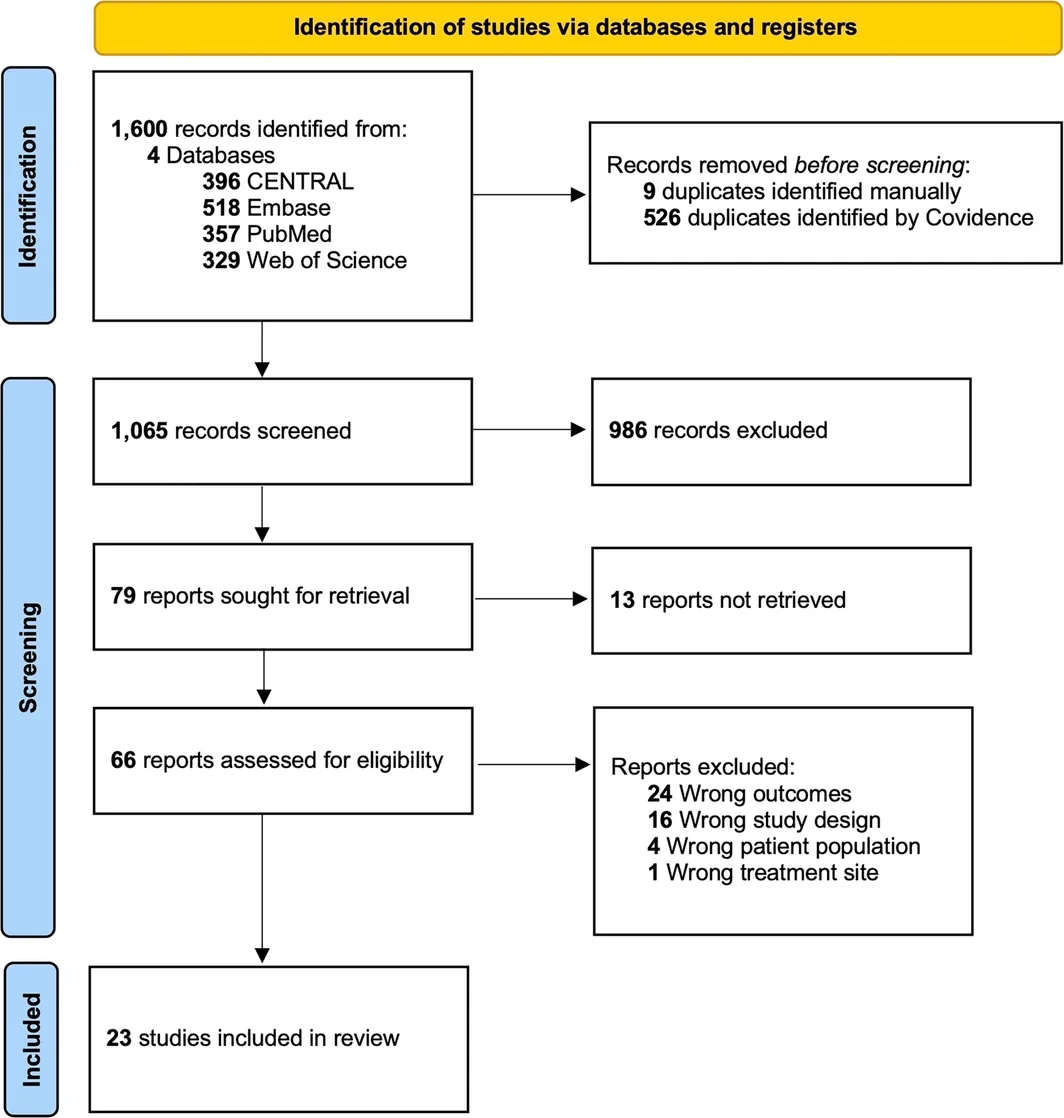
PRISMA flow diagram detailing the study selection process.

**TABLE 1:** Overview of Study Characteristics.

Authors	Study Design	*N*	Interventions	Protocol^a^
Light vs. Control				
Ding et al. (2022)	Split‐face RCT	11	Blue light vs. No treatment	Biweekly for 4 weeks (W1–W4)
Kwon et al. (2013)	Parallel‐group RCT	18 vs. 17	Blue–red LED device vs. Sham	Twice daily for 4 weeks (56 sessions total, W1–W4) FU at W8 and W12
Zane et al. (2008)	Single‐blinded, intrapatient, NRCT	15	Red light vs. No treatment	Biweekly for 4 weeks (W1–W4) FU at W16
Light vs. Light				
Wang et al. (2013)	Single‐blinded, multiarm nonrandomized trial	12 vs. 13 vs. 11	4 IPL + FCL (Group A) vs. 5 IPL + FCL (Group B) vs. 6 IPL + FCL (Group C)	IPL: 4–6 sessions with 3–4‐week intervals FCL^b^: 2 sessions with 8–12‐week intervals FU: 4–8 weeks after final IPL session; 12 and 24 weeks after last FCL session
PDT vs. Control				
Kwon et al. (2016)	Double‐blind parallel‐group RCT	23 vs. 23	ALA‐bu cream + daylight vs. VEH cream + daylight	Every other day for 12 weeks (W1–W12)
PDT vs. Light				
Akaraphanth et al. (2007)	Open‐label, split‐face NRCT	20	ALA + blue light vs. Blue light	4 sessions with 1‐week intervals (W1–W4) FU at W8 and W12
Horfelt et al. (2009)	Open‐label, split‐face, NRCT	23	MAL + red light vs. Red light	1 session (W0) FU at W1, W10, and W20
Na et al. (2011)	Split‐face RCT	14	IAA + green light vs. Control gel + green light	3 sessions with 2‐week intervals (W0–W4) FU at W6
Song et al. (2014)	Split‐face RCT	24	Chlorophyll‐*a* + blue–red LED vs. Blue–red LED	Twice weekly for 4 weeks (8 sessions, W1–W4) FU at W6
PDT vs. PDT				
Jang et al. (2011)	Single‐blind, split‐face, nonrandomized trial	34	ICG + 805 nm DL vs. IAA + green light	5 sessions with 1‐week intervals (W0–W4) FU at W5, W8, W12, and W16
Kim, Kim et al. (2023)	Double‐blind, split‐face randomized trial	31	SJW + green–red LED vs. IAA + green–red LED	4 sessions with 1‐week intervals (W0–W3) FU at W4 and W8
Oh et al. (2009)	Single‐blind, (parallel‐group) split‐face RCT	9 vs.11	(Short incubation ALA + IPL vs. Long incubation ALA + IPL) vs. IPL	3 sessions with 4‐week intervals (W0–W8) FU at W12, W16, and W20
Wangsuwan and Meephansan (2019)	Split‐face RCT	37	RT + blue light vs. ALA + blue light	4 sessions with 1‐week intervals (W0–W3) FU at W5 and W7
Laser vs. Control				
Jung et al. (2012)	Split‐face RCT	20	Dual‐mode 1064‐nm Nd:YAG laser + carbon suspension vs. No treatment	3 sessions with 2‐week intervals (W0–W4) FU at W8
Kesty and Goldberg (2020)	Parallel‐group RCT	10 vs. 10	650 μs 1064‐nm Nd:YAG laser vs. Sham	4 sessions (W0, W2, W4, W8) FU at W12
Laser vs. Light				
Kwon et al. (2019)	Split‐face RCT	25	1450‐nm DL + blue light vs. Blue light	3 sessions with 4‐week intervals (W0–W8) FU at W9 and W20
Laser vs. PDT				
Chen et al. (2023)	Parallel‐group RCT	12 vs. 12	QS 1064‐nm Nd:YAG laser + ALA + red light vs. ALA + red light	3 sessions with 4‐week intervals (W0–W8) FU at W12 and W24
Laser vs. Laser				
Kwon, Choi et al. (2018)	Split‐face RCT	25	Dual‐mode^c^ 1450‐nm DL vs. Stamp‐mode 1450‐nm DL	3 sessions with 4‐week intervals (W0–W8) FU at W9 and W20
RF vs. Control				
Choi et al. (2019)	Split‐face RCT	11	NMRF vs. No treatment	2 sessions with 2‐week intervals (W0–W2) FU at W4
RF vs. Laser				
Kim, Lee et al. (2023)	Split‐face RCT	25	FMR + FCL vs. FCL	3 sessions with 4‐week intervals (W0–W8) FU at W12 and W20
Kwon, Park et al. (2018)	Split‐face RCT	26	FMR vs. 1450‐nm DL	3 sessions with 4‐week intervals (W0–W8) FU at W9 and W20
Shin et al. (2012)	Single‐blind, split‐face RCT	20	FMR vs. FCL	1–2 sessions, 12 week FU
RF vs. RF				
Min et al. (2015)	Single‐blind, split‐face RCT	20	FMR vs. BR	2 sessions with 4 week intervals (W0–W4) FU at W8 and W12

Abbreviations: ALA, 5‐aminolevulinic acid; BR, bipolar radiofrequency; DL, diode laser; F, female; FCL, fractional carbon dioxide laser; FMR, fractional microneedling radiofrequency; FU, follow‐up; IAA, indole‐3‐acetic acid; ICG, indocyanine green; IPL, intense pulsed light; LED, light‐emitting diode; LP, long‐pulse mode; M, male; MAL, Methyl‐aminolevulinate; MTZ, microthermal treatment zone; N, sample size, Nd YAG, neodymium‐doped yttrium aluminum garnet; NMRF, nonablative monopolar radiofrequency; NRCT, nonrandomized control trial; PDT, photodynamic therapy; QS, Q‐switch mode; RCT, randomized control trial; RF, radiofrequency; RT, riboflavin‐tryptophan; SJW, St. John's Wort; W, week.

^a^ Follow‐up after final treatment session.

^b^ FCL treatment began 1–2 months after last IPL treatment.

^c^ Dual‐mode consists of stamp mode and moving mode. Stamp mode is the typical mode used with the 1450‐nm diode laser.

### 3.2 Study characteristics and demographics

Of the 23 studies included, 18 were randomized trials and five were nonrandomized, involving 559 patients (218 males and 307 females). The average participant age was 24.2 years (range 12–52 years). Acne severity varied from mild to severe, predominantly mild‐to‐moderate. Fitzpatrick skin types ranged from I to VI, mostly types III and IV. The studies were conducted in Asia, Europe, and North America. CSL measurements were obtained from 510 participants, primarily on the cheeks. Details are presented in Table 2 and Table S1 (Supplementary Material).

**TABLE 2:** Mechanism of Sebum Reduction for Treatments Reviewed.

Treatment	Sebum Reduction Mechanism
Light therapy	
Blue light	Targets and activates coproporphyrin produced by *C. acnes* to produce ROS that destroy *C. acnes*. Elimination of *C. acnes* decreases lipogenesis within SGs.
Red light	Wavelength penetrates up to 2 mm into the skin, reaching the SGs. Exerts anti‐inflammatory effects by inducing cytokine release from macrophages and photothermolysis of SGs.
Green light	Targets and activates coproporphyrin produced by *C. acnes* to produce ROS that destroy *C. acnes*. Elimination of *C. acnes* decreases lipogenesis within SGs.
Blue/red LED	630‐nm red light suppresses lipid synthesis in human sebocytes, whereas 415‐nm blue light inhibits sebocyte proliferation.
IPL	Selective photothermolysis of blood vessels supplying SGs. Reduction of SG size and/or function.
Daylight	Contains both red and blue light. Targets and activates porphyrins produced by *C. acnes* to produce ROS that destroy *C. acnes*. Elimination of *C. acnes* decreases lipogenesis within SGs.
PDT	
ALA	Topical 5‐ALA preferentially accumulates in SGs. Upon photoactivation, converts to PpIX causing phototoxic damage to SGs, reducing sebum, eradicating *C. acnes*, and decreasing follicular obstruction.
ALA‐bu	Decrease in pro‐inflammatory cytokines may reduce lipogenesis without significantly affecting SGs. Downregulation of PPAR‐γ could also contribute to sebum suppression.
MAL	Upon photoactivation, it converts to PpIX causing phototoxic damage to SGs, reducing sebum, eradicating *C. acnes*, and decreasing follicular obstruction.
RT	Activates tryptophan upon light exposure which generates ROS, reducing *C. acnes* and sebum.
ICG	Quickly accumulates in SGs. Generates ROS directly upon light exposure, reducing *C. acnes* and sebum. NIR radiation penetrates deeply into the skin to reach the SGs.
IAA	Eliminates *C. acnes*, which is known to increase lipogenesis within the SGs (18 Na).
Chlorophyll‐*a*	Generates ROS directly upon light exposure causing destruction to the SGs, reducing sebum and eradicating *C. acnes*.
SJW	Generation of ROS that target the SGs and eliminate *C. acnes*, reducing sebum and acne lesions.
Laser therapy	
1450‐nm DL	Target water to heat and destroy SGs and associated structures, reducing SG activity, sebum production, and acne lesions.
1064‐nm Nd:YAG laser	Target water to heat and destroy SGs and associated structures, reducing SG activity and size, sebum production, and acne lesions.
FCL	Creates tissue ablation zones in epidermis and part of dermis with surrounding coagulation, causing photothermolysis of SGs.
RF therapy	
NMRF	Targets water in the skin surrounding SGs, causing selective electrothermolysis of the glands.
BR	Targets water in the skin surrounding SGs, causing selective electrothermolysis of the glands.
FMR	Microneedling combined with BR leads to deeper delivery of thermal energy to the SGs causing greater destruction.

Abbreviations: ALA, 5‐aminolevulinic acid; BR bipolar radiofrequency; C. acnes, Cutibacterium acnes; CSL, casual sebum level; DL, diode laser; FCL, fractional carbon dioxide laser; FMR, fractional microneedling radiofrequency; IAA, indole‐3‐acetic acid; ICG, indocyanine green; IPL, intense pulsed light; LED, light‐emitting diode; MAL, methyl‐aminolevulinate; Nd YAG, neodymium‐doped yttrium aluminum garnet; NMRF, nonablative monopolar radiofrequency; PDT, photodynamic therapy; PPAR‐γ, Peroxisome proliferator‐activated receptor‐γ; PpIX, Protoporphyrin IX; RF, radiofrequency; ROS, reactive oxygen species; RT, riboflavin‐tryptophan; SG, sebaceous gland; SJW, St. John's Wort.

### 3.3 Risk of Bias Assessment

The risk of bias assessment is shown in Figure 2A,B for randomized trials, and Figure 3A,B for nonrandomized trials.

**FIGURE 2:**
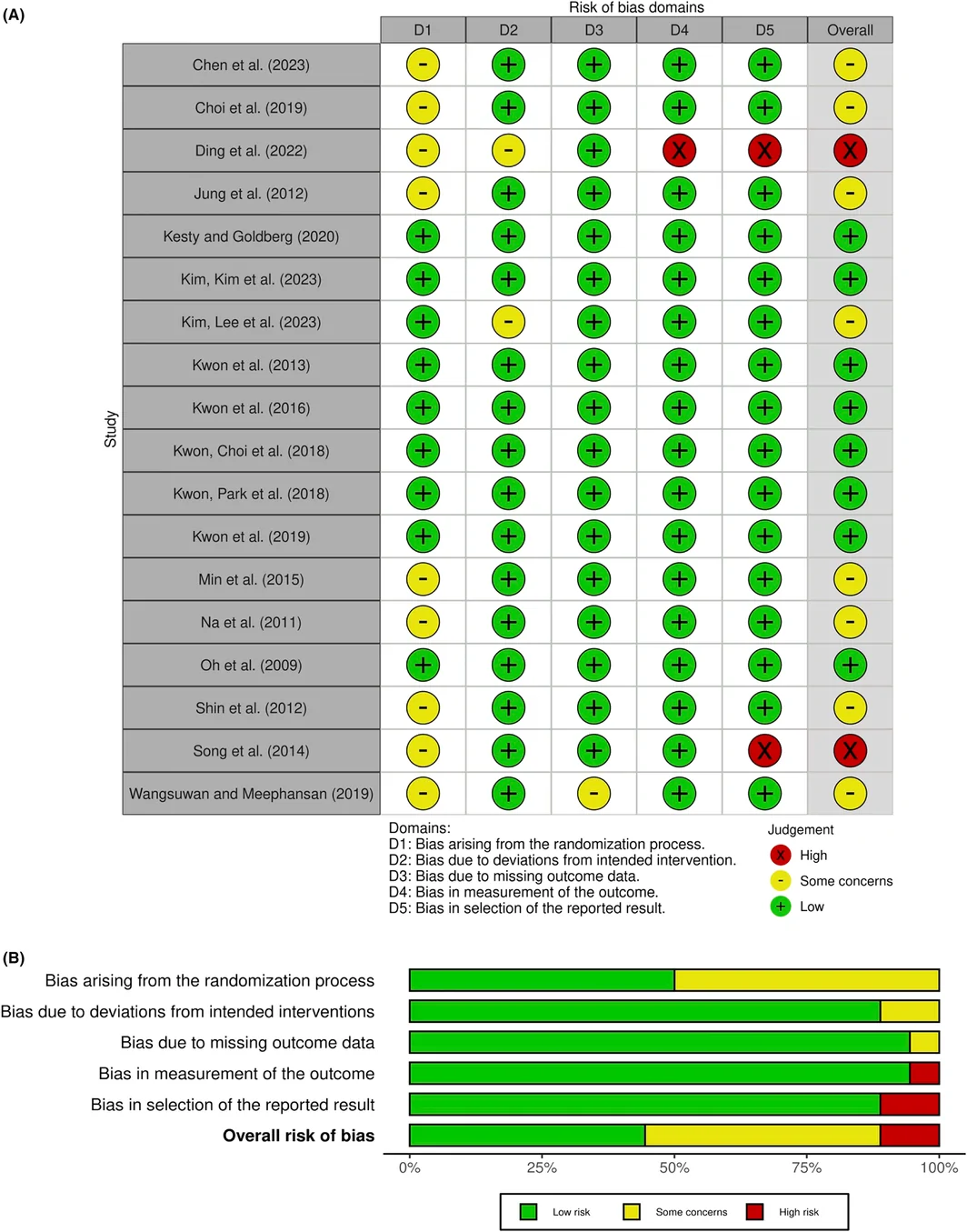
Risk of bias assessment for randomized trials using RoB 2.0: (A) Traffic light plot; (B) Summary plot.

**FIGURE 3:**
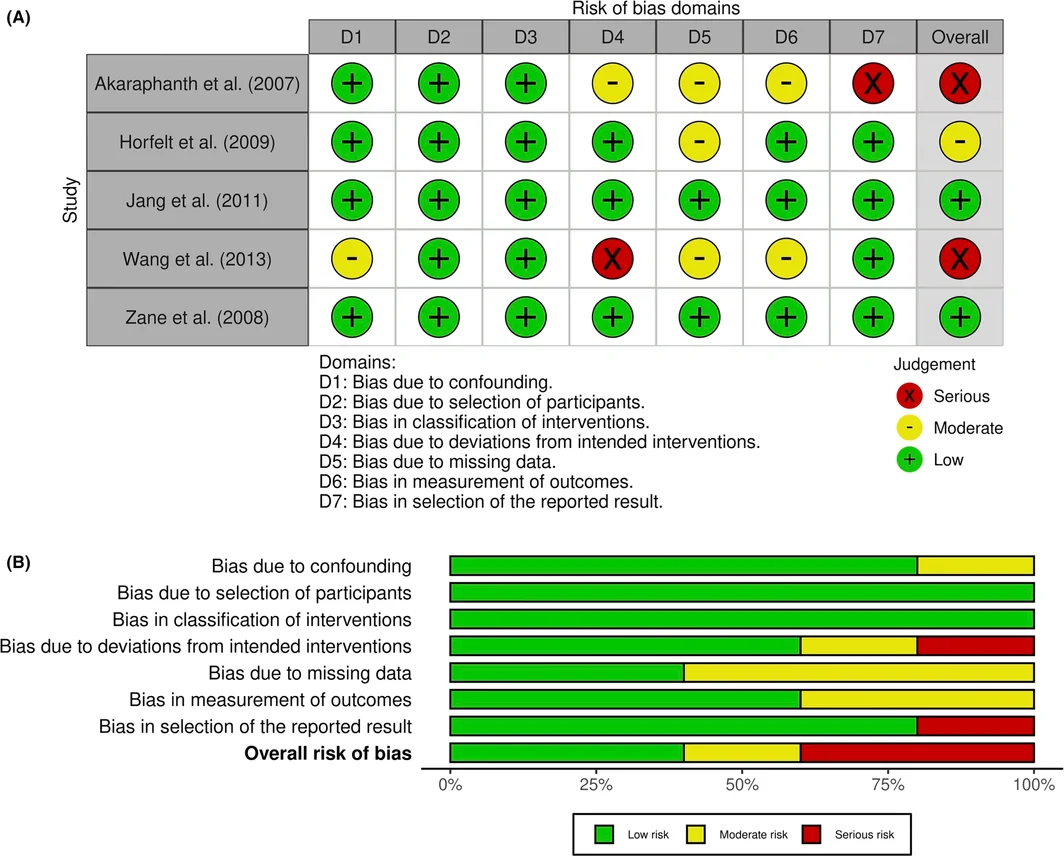
Risk of bias assessment for nonrandomized trials using ROBINS‐I: (A) Traffic light plot; (B) Summary plot.

### 3.4 Summary of study findings

Table S2 provides detailed results for each study.

#### 3.4.1 Light versus Control

Ding et al.^1^ observed a 60.9% CSL reduction (*p* = 0.008) after eight blue light treatments. Kwon et al.^10^ reported a 19.9% CSL decrease after blue–red LED treatment at 12 weeks (*p* < 0.001). Zane et al.^11^ found a 43% CSL reduction after eight red light treatments at the 12‐week follow‐up (*p* < 0.05). Control sites showed no reduction, and no quantitative data was provided.^11^

#### 3.4.2 Light versus Light

Wang et al.^12^ evaluated the efficacy of combining IPL treatments with fractional carbon dioxide laser (FCL) sessions across three groups, differentiated by the number of IPL sessions received: four, five, or six (Groups A, B, and C). Posttreatment, all groups displayed significant reductions in CSL after IPL (A, 19.6%; B, 23.8%; C, 16.0%) and 12 weeks after FCL, except for Group C, where the reduction was not statistically significant. No significant differences were found between the groups.^12^

#### 3.4.3 PDT versus Control

Kwon et al.^13^ found a gradual, significant CSL reduction of 32.6% at 12 weeks with ALA‐bu cream and daylight PDT compared to baseline (*p* < 0.05). Treatment also outperformed controls (*p* < 0.05).^13^

#### 3.4.4 PDT versus Light

Na et al.^14^ found IAA with green light resulted in a 34.1% CSL reduction (*p* < 0.05), while green light alone showed a 27.7% nonsignificant decrease.

Song et al.^15^ reported Chlorophyll‐a with blue–red LED significantly outperformed LED alone, with a 37.4% reduction at week 6 compared to 16.5% (*p* < 0.001). PDT also led to a faster CSL reduction, which was significant from 2 weeks onwards (*p* < 0.05).^15^

However, Akaraphanth et al.^16^ and Horfelt et al.^17^ found no significant CSL reduction with 5‐aminolevulinic acid (ALA)‐PDT with blue light and methyl‐aminolevulinate (MAL)‐PDT with red light, respectively. Additionally, blue and red light monotherapy did not significantly reduce CSL.^16^, ^17^

#### 3.4.5 PDT versus PDT

Jang et al.^18^ compared indocyanine green (ICG)‐PDT with 805‐nm diode laser (DL) to IAA‐PDT with green light, noting 42.3% and 33.9% CSL reductions at week 5, respectively. Both treatments showed comparable efficacy.^18^

At the 4‐week follow‐up, Kim et al.^19^ found that St. John's Wort (SJW)‐PDT and IAA‐PDT using green–red LED significantly reduced CSL compared to baseline by 27.2% (*p* = 0.022) and 26.9% (*p* < 0.001), respectively. There were no significant differences between them.^19^

Oh et al.^20^ compared the effect of incubation length on the effectiveness of 20% ALA‐PDT using IPL. All groups showed significant CSL reductions at the 12‐week follow‐up: 31.5% for short incubation, 46.7% for long incubation, and 37.5% for IPL alone (*p* < 0.001). There were no significant differences between the groups.^20^

Wangsuwan and Meephansan^21^ compared riboflavin‐tryptophan (RT) gel to 13% ALA using blue light. Both treatments significantly reduced CSL starting from the first treatment, with RT‐PDT and ALA‐PDT showing reductions of 54.4% and 56.6%, respectively, at the 4‐week follow‐up (*p* < 0.001). No significant differences were observed between the treatments.^21^

#### 3.4.6 Laser versus Control

In assessing laser treatment efficacy compared to controls, Jung et al.^22^ found a significant CSL reduction after three treatments (*p* = 0.001) and at follow‐up (*p* < 0.05) with dual‐mode 1064‐nm Nd:YAG laser combined with carbon suspension. There was a 27.7% CSL reduction at follow‐up compared to baseline (*p* = 0.025).^22^

Kesty and Goldberg^23^ evaluated the 650 μs 1064‐nm Nd:YAG laser versus a sham device, with the laser group showing a larger CSL decrease from baseline at the 4‐week follow‐up (18% vs. 9%).

#### 3.4.7 Laser versus Light

Kwon et al.^24^ compared the 1450‐nm DL combined with blue light to blue light alone. Combination therapy significantly reduced CSL compared to baseline and blue light monotherapy from the first treatment onwards, showing a 41% reduction versus an 11.4% increase with monotherapy at the 12‐week follow‐up (*p* < 0.05).^24^

#### 3.4.8 Laser versus PDT

Chen et al.^25^ compared the efficacy of 2% ALA with red light on skin pretreated with the Q‐switch 1064‐nm Nd:YAG laser to ALA‐PDT alone. Combined therapy significantly reduced CSL compared to monotherapy at the end of treatment (27.7% vs. 9.2%) and week 12 (37.5% vs. 16.3%) (*p* < 0.05).^25^

#### 3.4.9 Laser versus Laser

Kwon et al.^26^ compared the efficacy of dual‐mode versus stamp‐mode using the 1450‐nm DL. Both methods significantly reduced CSL after one treatment session (*p* < 0.05). By week 20, the dual‐mode laser achieved a 47.8% reduction in CSL (*p* < 0.05). The conventional laser side had a CSL increase at week 20 compared to week 9, with reductions of 21.7% versus 28%; however, this was still significantly lower than baseline (*p* < 0.05). The dual‐mode laser was significantly more effective than stamp‐mode from the second treatment onwards (*p* < 0.05).^26^

#### 3.4.10 RF versus Control

Choi et al.^27^ found a significant 23.1% reduction in CSL after nonablative monopolar radiofrequency (NMRF) laser treatment compared to the control side at the 2‐week follow‐up (*p* < 0.017).

#### 3.4.11 RF versus Laser

Kim et al.^28^ found that combining fractional microneedle radiofrequency (FMR) with FCL significantly reduced CSL by 12.5% compared to FCL alone, which showed a 13.9% CSL increase (*p* < 0.05).

Kwon et al.^29^ observed a significant CSL reduction with both 1450‐nm DL and FMR after two sessions compared to baseline. By week 20, FMR and DL showed 33.9% and 34.2% reductions, respectively (*p* < 0.05). Notably, FMR maintained better long‐term CSL reduction compared to DL (*p* < 0.05), which displayed reduced effectiveness at week 20 compared to week 9.^29^

Shin et al.^30^ found that both FMR and FCL treatment significantly reduced CSL at follow‐up compared to baseline, with reductions of 30.9% and 31%, respectively (*p* < 0.05). No significant differences were observed between the groups.^30^

#### 3.4.12 RF versus RF

Min et al.^31^ compared FMR to bipolar radiofrequency (BR). At the 4‐week follow‐up, FMR significantly reduced CSL by 63.5% from baseline (*p* = 0.002), whereas the reduction with BR treatment was not significant (34.2%). At the final follow‐up, FMR reached a 65.4% reduction, whereas BR worsened to 30.9%. FMR significantly outperformed BR at both follow‐up visits (*p* < 0.001).^31^

## 4 DISCUSSION

Previous research has shown that a 30%–50% reduction in sebum is needed to achieve a 50% decrease in acne severity metrics.^32^ However, the effectiveness of traditional treatments in reducing sebum levels varies. Isotretinoin can achieve a sebum reduction of 60%–90%, while hormonal therapies may result in up to a 75% decrease.^32^ Topicals, such as adapalene and benzoyl peroxide, show modest effects, with adapalene causing a 0.2%–7% decrease^33^ and benzoyl peroxide showing variable impacts ranging from a 10%–25% increase^33^ to a 20% decrease^34^ in sebum production.

Regarding EBDs, PDT reduced sebum levels by 30%–40% in most studies, regardless of the photosensitizer used. RF devices also provided consistent reductions of 30%–35%. Laser therapy was typically less effective, except for 1450‐nm DL, which achieved 40%–48% reductions. However, light therapy exhibits more variable outcomes and studies had a higher risk of bias. This may be due to rapid *C. acnes* reproduction in overactive SGs after light therapy.^35^

All therapies outperformed controls. PDT has excelled over light therapy. Laser therapy, particularly 1450‐nm DL, showed greater CSL reduction than light or PDT. FMR, alone or with laser, also outperformed laser monotherapy. Although these therapies did not achieve the reductions in sebum production seen with isotretinoin or hormonal therapies, most treatments still lead to a significant decrease.

When using EBDs for AV, selecting the correct treatment parameters is crucial. Effective treatment often requires high doses, prolonged contact, and high‐fluence exposure; however, some exceptions exist. For PDT, recent studies suggest that extended incubation time^20^, ^21^, ^25^ and higher concentrations^21^, ^25^ may not be necessary to reduce CSL and treat acne lesions, highlighting the need for further research to optimize protocols.

Several factors must also be considered when measuring CSL. Sebum levels are highly sensitive to the external environment and can fluctuate rapidly, making accurate measurements challenging.^2^ Allowing a 30–60 min acclimatization period before measurements can help control these variables.^2^ While only three studies specified an acclimatization period,^1^, ^12^, ^16^ most instructed participants not to wash their faces for 5–6 h premeasurement. Eight studies did not describe the measurement methods.

Furthermore, there is debate regarding the best location for sebum measurements. The T‐zone (forehead, nose, chin) has the highest concentration of SGs on the face and exhibits significantly higher CSLs than the U‐zone (cheeks).^2^ The T‐zone has been recommended for measurements; however, some argue that measuring areas with high SG activity may lead to inaccuracies and greater variability. Recent research suggests that the U‐zone reliably reflects facial sebum levels and exhibits lower but stable sebum content, whereas the T‐zone, especially the nose, exhibits significant fluctuations, making it less suitable for accurate measurements.^2^ Of the trials in this review that specified measurement location, 61% solely used cheeks.

The T‐zone requires a stricter degreasing protocol than the U‐zone. Forehead degreasing involves washing with detergent followed by alcohol wipes, whereas cheeks only require mild soap.^5^ Additionally, a 5–6 h measurement delay postwashing provides more accurate results for the forehead.^5^ This may explain the lack of efficacy on the forehead in Akaraphanth et al.'s^16^ study, which reported a brief acclimatization period postwash. Despite these considerations, the Sebumeter's simplicity and quick operation are beneficial and reduce risk of measurement errors.^5^

A gap exists in studies measuring CSL in AV, especially for EBDs. Two‐arm studies are absent for devices such as the 585–595‐nm pulsed dye laser (PDL), 1540‐nm Er:glass laser, 1320‐nm Nd:YAG laser, potassium titanyl‐phosphate (KTP) laser, and others. Additionally, there is a lack of data on the 1726‐nm laser, and given its capacity to selectively target and destroy SGs,^36^, ^37^, ^38^ future research should incorporate CSL measurements to comprehensively assess the laser's efficacy in reducing sebum. This evaluation could help broaden the potential of this laser for other conditions characterized by overactive SGs.

Ultimately, when selecting an EBD for AV treatment, it is crucial to prioritize proper hydration and sebum control to maintain the health of acne‐prone skin. Clinicians often focus primarily on reducing acne lesions, overlooking the importance of skincare in treatment efficacy. Acne treatments can lead to dry skin, compromised barrier function, and decreased hydration.^39^ With the growing use of EBDs for AV, understanding their impact on skin health is essential.

## 5 LIMITATIONS

Regarding limitations, 52% of studies lacked numerical CSL data, relying on graphical representations, potentially introducing inaccuracies despite our standardized extraction approach. Data variability, which is crucial for result reliability, was rarely reported. Despite contacting the authors, no response was received. Given the inherent variability of sebum output, detailed variability reporting is essential.

Geographically, 87% of studies involved Asian populations, with 65% conducted in Korea, suggesting limited diversity. Future research should be conducted in diverse geographical locations.

## 6 CONCLUSION

Reliable noninvasive methods for assessing skin biophysical parameters such sebum, offer objective insights into skin health and treatment efficacy. When evaluating EBD therapies for AV, PDT and RF consistently reduced CSLs, whereas laser therapy showed slightly lower but significant reductions. Light therapy yielded inconsistent results and may be biased. Despite not reaching the sebum reduction levels of isotretinoin or hormonal treatments, EBDs still led to notable CSL reductions.

The review highlights challenges in CSL measurement, stressing the importance of site, environment, and methodology. Standardized protocols are crucial given the diverse measurement techniques and documentation gaps. Further research is necessary to understand the impact of EBDs on skin health in AV patients, enabling personalized treatment, minimizing side effects, and ensuring proper postprocedural care.

## AUTHOR CONTRIBUTIONS

DJ and JH contributed to the design and implementation of the research, DJ, YA, AP to the analysis of the results and to the writing of the manuscript. DG supervised the project and reviewed the paper for submission.

## CONFLICT OF INTEREST STATEMENT

The authors declare that they have no affiliations with or involvement in any organization or entity with any financial interest in the subject matter or materials discussed in this manuscript.

## ETHICS STATEMENT

None.

## Supporting information


Appendix S1.

Data S1.

## Data Availability

Data sharing is not applicable to this article as no new data were created or analyzed in this study.

## References

[1] Ding W , Hu Y , Yu X , He C , Tian Y . Analysis on the difference of skin surface lipids during blue light therapy for acne by lipidomics. Biomed Opt Express. 2022;13(6):3434‐3445. doi:10.1364/BOE.452614 PMC9208599 35781946

[2] Liu Y , Jiang W , Tang Y , et al. An optimal method for quantifying the facial sebum level and characterizing facial sebum features. Skin Res Technol. 2023;29(9):e13454. doi:10.1111/srt.13454 PMC10483949 37753695

[3] Del Rosso JQ , Kircik L . The primary role of sebum in the pathophysiology of acne vulgaris and its therapeutic relevance in acne management. J Dermatolog Treat. 2024;35(1):2296855. doi:10.1080/09546634.2023.2296855 38146664

[4] Sigurdsson V , Knulst AC , van Weelden H . Phototherapy of acne vulgaris with visible light. Dermatology. 1997;194(3):256‐260. doi:10.1159/000246114 9187844

[5] Sakamoto FH , Lopes JD , Anderson RR . Photodynamic therapy for acne vulgaris: a critical review from basics to clinical practice: part I. Acne vulgaris: when and why consider photodynamic therapy? J Am Acad Dermatol. 2010;63(2):183‐194. doi:10.1016/j.jaad.2009.09.056 20633796

[6] Angel S , Boineau D , Dahan S , Mordon S . Treatment of active acne with an Er:glass (1.54 microm) laser: a 2‐year follow‐up study. J Cosmet Laser Ther. 2006;8(4):171‐176. doi:10.1080/14764170600915985 19839169

[7] Sterne JAC , Savović J , Page MJ , et al. RoB 2: a revised tool for assessing risk of bias in randomised trials. BMJ. 2019;366:l4898. doi:10.1136/bmj.l4898 31462531

[8] Sterne JAC , Hernán MA , Reeves BC , et al. ROBINS‐I: a tool for assessing risk of bias in non‐randomized studies of interventions. BMJ. 2016;355:i4919. doi:10.1136/bmj.i4919 PMC5062054 27733354

[9] McGuinness LA , Higgins JPT . Risk‐of‐bias VISualization (robvis): an R package and shiny web app for visualizing risk‐of‐bias assessments. Res Synth Methods. 2021;12(1):55‐61. doi:10.1002/jrsm.1411 32336025

[10] Kwon HH , Lee JB , Yoon JY , et al. The clinical and histological effect of home‐use, combination blue‐red LED phototherapy for mild‐to‐moderate acne vulgaris in Korean patients: a double‐blind, randomized controlled trial. Br J Dermatol. 2013;168(5):1088‐1094. doi:10.1111/bjd.12186 23278295

[11] Zane C , Capezzera R , Pedretti A , Facchinetti E , Calzavara‐Pinton P . Non‐invasive diagnostic evaluation of phototherapeutic effects of red light phototherapy of acne vulgaris. Photodermatol Photoimmunol Photomed. 2008;24(5):244‐248. doi:10.1111/j.1600-0781.2008.00368.x 18811865

[12] Wang B , Wu Y , Luo YJ , et al. Combination of intense pulsed light and fractional CO(2) laser treatments for patients with acne with inflammatory and scarring lesions. Clin Exp Dermatol. 2013;38(4):344‐351. doi:10.1111/ced.12010 23551214

[13] Kwon HH , Moon KR , Park SY , Yoon JY , Suh DH , Lee JB . Daylight photodynamic therapy with 1.5% 3‐butenyl 5‐aminolevulinate gel as a convenient, effective and safe therapy in acne treatment: a double‐blind randomized controlled trial. J Dermatol. 2016;43(5):515‐521. doi:10.1111/1346-8138.13191 26660491

[14] Na JI , Kim SY , Kim JH , Youn SW , Huh CH , Park KC . Indole‐3‐acetic acid: a potential new photosensitizer for photodynamic therapy of acne vulgaris. Lasers Surg Med. 2011;43(3):200‐205. doi:10.1002/lsm.21029 21412803

[15] Song BH , Lee DH , Kim BC , et al. Photodynamic therapy using chlorophyll‐a in the treatment of acne vulgaris: a randomized, single‐blind, split‐face study. J Am Acad Dermatol. 2014;71(4):764‐771. doi:10.1016/j.jaad.2014.05.047 24930587

[16] Akaraphanth R , Kanjanawanitchkul W , Gritiyarangsan P . Efficacy of ALA‐PDT vs blue light in the treatment of acne. Photodermatol Photoimmunol Photomed. 2007;23(5):186‐190. doi:10.1111/j.1600-0781.2007.00303.x 17803597

[17] Hörfelt C , Stenquist B , Larkö O , Faergemann J , Wennberg AM . Photodynamic therapy for acne vulgaris: a pilot study of the dose‐response and mechanism of action. Acta Derm Venereol. 2007;87(4):325‐329. doi:10.2340/00015555-0243 17598035

[18] Jang MS , Doh KS , Kang JS , Jeon YS , Suh KS , Kim ST . A comparative split‐face study of photodynamic therapy with indocyanine green and indole‐3‐acetic acid for the treatment of acne vulgaris. Br J Dermatol. 2011;165(5):1095‐1100. doi:10.1111/j.1365-2133.2011.10472.x 21692772

[19] Kim BR , Kim M , Na JI , Huh CH , Shin JW . A randomized Split‐face study of photodynamic therapy with St. John's wort and Indole‐3‐acetic acid for the treatment of acne. Dermatologic Surg. 2023;49(5):483‐488. doi:10.1097/DSS.0000000000003742 36946749

[20] Oh SH , Ryu DJ , Han EC , Lee KH , Lee JH . A comparative study of topical 5‐aminolevulinic acid incubation times in photodynamic therapy with intense pulsed light for the treatment of inflammatory acne. Dermatologic Surg. 2009;35(12):1918‐1926. doi:10.1111/j.1524-4725.2009.01315.x 19708873

[21] Wangsuwan S , Meephansan J . Comparative study of photodynamic therapy with riboflavin‐tryptophan gel and 13% 5‐Aminolevulinic acid in the treatment of mild to moderate acne vulgaris. Clin Cosmet Investig Dermatol. 2019;12:805‐814. doi:10.2147/CCID.S227737 PMC6842286 31807046

[22] Jung JY , Hong JS , Ahn CH , Yoon JY , Kwon HH , Suh DH . Prospective randomized controlled clinical and histopathological study of acne vulgaris treated with dual mode of quasi‐long pulse and Q‐switched 1064‐nm Nd:YAG laser assisted with a topically applied carbon suspension. J Am Acad Dermatol. 2012;66(4):626‐633. doi:10.1016/j.jaad.2011.08.031 22033354

[23] Kesty K , Goldberg DJ . 650 usec 1064nm Nd:YAG laser treatment of acne: a double‐blind randomized control study. J Cosmet Dermatol. 2020;19(9):2295‐2300. doi:10.1111/jocd.13480 32447830

[24] Kwon HH , Choi SC , Jung JY , Bae Y , Park GH . A novel combined light‐based treatment of acne vulgaris with 1,450‐nm diode laser and 450‐nm blue light. Dermatologic Surg. 2019;45(9):1147‐1154. doi:10.1097/DSS.0000000000001815 30789515

[25] Chen YT , Lin YJ , Chang CC , et al. The quantitative analysis of low‐concentration (2%) ALA‐PDT assisted with Q‐switch 1064‐nm Nd:YAG laser for acne vulgaris treatment. Lasers Med Sci. 2023;38(1):236. doi:10.1007/s10103-023-03896-x 37843672

[26] Kwon HH , Choi SC , Jung JY , Bae YI , Park GH . Comparison of novel dual mode vs conventional single pass of a 1450‐nm diode laser in the treatment of acne vulgaris for Korean patients: a 20‐week prospective, randomized, split‐face study. J Cosmet Dermatol. 2018;17(6):1063‐1068. doi:10.1111/jocd.12788 30225903

[27] Choi J , Hwang SH , Yang J , Lee G . Using a nonablative monopolar radiofrequency laser in the treatment of acne vulgaris. Dermatol Sin. 2019;37(2):77‐81. doi:10.4103/ds.ds_4_18

[28] Kim J , Lee SG , Choi S , et al. Combination of fractional microneedling radiofrequency and ablative fractional laser versus ablative fractional laser alone for acne and acne scars. Yonsei Med J. 2023;64(12):721‐729. doi:10.3349/ymj.2023.0234 PMC10681823 37992744

[29] Kwon HH , Park HY , Choi SC , Bae Y , Jung JY , Park GH . Novel device‐based acne treatments: comparison of a 1450‐nm diode laser and microneedling radiofrequency on mild‐to‐moderate acne vulgaris and seborrhoea in Korean patients through a 20‐week prospective, randomized, split‐face study. J Eur Acad Dermatol Venereol. 2018;32(4):639‐644. doi:10.1111/jdv.14714 29178495

[30] Shin JU , Lee SH , Jung JY , Lee JH . A split‐face comparison of a fractional microneedle radiofrequency device and fractional carbon dioxide laser therapy in acne patients. J Cosmet Laser Ther. 2012;14(5):212‐217. doi:10.3109/14764172.2012.720023 23016530

[31] Min S , Park SY , Yoon JY , Suh DH . Comparison of fractional microneedling radiofrequency and bipolar radiofrequency on acne and acne scar and investigation of mechanism: comparative randomized controlled clinical trial. Arch Dermatol Res. 2015;307(10):897‐904. doi:10.1007/s00403-015-1601-z 26472097

[32] Janiczek‐Dolphin N , Cook J , Thiboutot D , Harness J , Clucas A . Can sebum reduction predict acne outcome? Br J Dermatol. 2010;163(4):683‐688. doi:10.1111/j.1365-2133.2010.09878.x 20518779

[33] Stinco G , Bragadin G , Trotter D , Pillon B , Patrone P . Relationship between sebostatic activity, tolerability and efficacy of three topical drugs to treat mild to moderate acne. J Eur Acad Dermatol Venereol. 2007;21(3):320‐325. doi:10.1111/j.1468-3083.2006.01914.x 17309452

[34] Kaewsanit T , Chakkavittumrong P , Waranuch N . Clinical comparison of topical 2.5% benzoyl peroxide plus 5% niacinamide to 2.5% benzoyl peroxide alone in the treatment of mild to moderate facial acne vulgaris. J Clin Aesthet Dermatol. 2021;14(6):35‐41. PMC8594539 34804354

[35] Saint‐Leger D , Cohen E . Practical study of qualitative and quantitative sebum excretion on the human forehead. Br J Dermatol. 1985;113(5):551‐557. doi:10.1111/j.1365-2133.1985.tb02378.x 4063191

[36] Goldberg D , Kothare A , Doucette M , et al. Selective photothermolysis with a novel 1726 nm laser beam: a safe and effective solution for acne vulgaris. J Cosmet Dermatol. 2023;22(2):486‐496. doi:10.1111/jocd.15602 36576854

[37] Goldberg DJ , Andriessen A , Bhatia AC , et al. Treatment of mild to severe acne with 1726 nm laser: a safe alternative to traditional acne therapies. J Cosmet Dermatol. 2023;22(11):3026‐3032. doi:10.1111/jocd.15964 37596781

[38] Alexiades M , Kothare A , Goldberg D , Dover JS . Novel 1726 nm laser demonstrates durable therapeutic outcomes and tolerability for moderate‐to‐severe acne across skin types. J Am Acad Dermatol. 2023;89(4):703‐710. doi:10.1016/j.jaad.2023.05.085 37328000

[39] Schachner LA , Alexis AF , Andriessen A , et al. Insights into acne and the skin barrier: optimizing treatment regimens with ceramide‐containing skincare. J Cosmet Dermatol. 2023;22(11):2902‐2909. doi:10.1111/jocd.15946 37605504

