# Supplementary material for: The impact of energy‐based devices on sebum in acne vulgaris: A systematic review
Source: J Cosmet Dermatol. 2024 Aug 11;23(10):3066–77. doi: 10.1111/jocd.16466 (PMC13649100; doi:10.1111/jocd.16466)
Supplement: Supplementary file 1 [file JOCD-23--s001.docx]

**Appendix**

**1.0 Search Strategy**

**1.1 Embase**

(‘acne vulgaris’/exp OR ‘acne’) AND ('laser'/exp OR 'laser' OR 'lasers'/exp OR 'lasers' OR 'phototherapy'/exp OR 'phototherapy' OR 'light'/exp OR 'light' OR 'laser therapy'/exp OR 'laser therapy' OR 'laser therapies' OR 'photodynamic therapy'/exp OR 'photodynamic therapy' OR 'pdt' OR 'light therapy'/exp OR 'light therapy' OR 'radiofreqency'/exp OR 'radiofrequency' OR ‘rf’ OR 'light therapies' OR ‘energy-based device’) AND ('sebum'/exp OR 'sebum' OR 'sebum secretion'/exp OR 'sebum secretion' OR 'sebum excretion'/exp OR 'sebum excretion' OR ‘sebumeter’ OR ‘casual sebum level’ OR ‘sebum output’ OR ‘sebaceous glands’)

**1.2 MEDLINE**

(“acne vulgaris” OR “acne”) AND (“laser” OR “lasers” OR “laser therapy” OR “photodynamic therapy” OR “pdt” OR “light” OR “light therapy” OR “phototherapy” OR “radiofrequency” OR “RF” OR “energy-based device”) AND (“sebum” OR “casual sebum level” OR “Sebumeter” OR “sebaceous glands” OR “sebum output”)

**1.3 Web of Science**

(“acne vulgaris” OR “acne”) AND (“laser” OR “lasers” OR “laser therapy” OR “photodynamic therapy” OR “pdt” OR “light” OR “light therapy” OR “phototherapy” OR “radiofrequency” OR “RF” OR “energy-based device”) AND (“sebum” OR “casual sebum level” OR “Sebumeter” OR “sebaceous glands” OR “sebum output”)

**1.4 CENTRAL**

(“acne vulgaris” OR “acne”) AND (“laser” OR “lasers” OR “laser therapy” OR “photodynamic therapy” OR “pdt” OR “light” OR “light therapy” OR “phototherapy” OR “radiofrequency” OR “RF” OR “energy-based device”) AND (“sebum” OR “casual sebum level” OR “Sebumeter” OR “sebaceous glands” OR “sebum output”)
