# Supplementary material for: The impact of energy‐based devices on sebum in acne vulgaris: A systematic review
Source: J Cosmet Dermatol. 2024 Aug 11;23(10):3066–77. doi: 10.1111/jocd.16466 (PMC13649100; doi:10.1111/jocd.16466)
Supplement: Supplementary file 2 [file JOCD-23--s002.docx]

**Supplementary Material**

**Table S1.** Study Demographics

| Authors | Country | Sex (M/F) | Age^a^ | Disease Severity | Fitzpatrick Skin Type |
| --- | --- | --- | --- | --- | --- |
| Light vs. Control | | | | | |
| Ding et al. (2022) | China | 4/7 | 25.3 ± 6.2 (19–43) | Mild-to-moderate | – |
| Kwon et al. (2013) | Korea | 13/5 vs. 11/6 | 22.5 ± 3.2 vs. 23.7 ± 2.6 (20–27) | Mild-to-moderate | III–V |
| Zane et al. (2008) | Italy | 0/15 | 22.4 (17–29) | Moderate | – |
| Light vs. Light | | | | | |
| Wang et al. (2013) | China | 11/26 | 24.1 ± 7.2 (21–35) | – | III–IV |
| PDT vs. Control | | | | | |
| Kwon et al. (2016) | Korea | 13/10 vs. 11/12 | 22.4 ± 2.4 vs. 23.1 ± 3.6 | Moderate | III–V |
| PDT vs. Light | | | | | |
| Akaraphanth et al. (2007) | Thailand | 4/16 | 23.3 (18–35) | Moderate-to-severe | – |
| Horfelt et al. (2009) | Sweden | 9/14 | 19–37 | Moderate-to-severe | I–III |
| Na et al. (2011) | Korea | – | – | – | – |
| Song et al. (2014) | Korea | 14/10 | 23.4 ± 3.5 (18–32) | Moderate-to-severe | III–IV |
| PDT vs. PDT | | | | | |
| Jang et al. (2011) | Korea | 12/22 | 23.1 (17–29) | Mild-to-moderate | III–V |
| Kim, Kim et al. (2023) | Korea | 18/13 | 31.7 ± 6.2 (25–52) | Mild-to-moderate | II–VI |
| Oh et al. (2009) | Korea | 3/6 vs. 1/9 | 23 ± 4.1 vs. 23 ± 5.5 (18–30) | Moderate-to-severe | III–IV |
| Wangsuwan and Meephansan (2019) | Thailand | 9/25 | 28.26 ± 8.4 | Mild-to-moderate | – |
| Laser vs. Control | | | | | |
| Jung et al. (2012) | Korea | 4/16 | 25.4 (19–34) | Mild-to-moderate | III–V |
| Kesty and Goldberg (2020) | United States | – | 12–40 | Moderate-to-severe | – |
| Laser vs. Light | | | | | |
| Kwon et al. (2019) | Korea | 11/14 | 21.6 ± 7.8 (18–39) | Mild-to-moderate | III–IV |
| Laser vs. PDT | | | | | |
| Chen et al. (2023) | Taiwan | 2/10 vs. 5/7 | 25.3 ± 7.6 vs. 23.8 ± 5.9 | Mild-to-moderate | III–IV |
| Laser vs. Laser | | | | | |
| Kwon, Choi et al. (2018) | Korea | 9/16 | 23.4 ± 7.8 (18–36) | Mild-to-moderate | III–V |
| RF vs. Control | | | | | |
| Choi et al. (2019) | Korea | 6/5 | 22.3 (16–27) | – | III–IV |
| RF vs. Laser | | | | | |
| Kim, Lee et al. (2023) | Korea | 16/9 | 24.1 ± 4.76 (19–36) | – | III–IV |
| Kwon, Park et al. (2018) | Korea | 10/16 | 23.4 ± 7.8 (19–37) | Mild-to-moderate | III–IV |
| Shin et al. (2012) | Korea | 11/9 | 23.6 (15–28) | Mild-to-moderate | III–IV |
| RF vs. RF | | | | | |
| Min et al. (2015) | Korea | 11/9 | 22.8 | Mild-to-moderate | III–IV |

Abbreviations: *ALA* 5-aminolevulinic acid, *BR* bipolar radiofrequency, *DL* diode laser, *F* female, *FCL* fractional carbon dioxide laser, *FMR* fractional microneedling radiofrequency, *FU* follow-up, *IAA* indole-3-acetic acid, *ICG* indocyanine green, *IPL* intense pulsed light, *LED* light-emitting diode, *LP* long-pulse mode, *M* male, *MAL* Methyl-aminolevulinate, *MTZ* microthermal treatment zone, *N* sample size, *Nd:YAG* neodymium-doped yttrium aluminum garnet, *NMRF* non-ablative monopolar radiofrequency, *NRCT* non-randomized control trial, *PDT* photodynamic therapy, *QS* Q-switch mode, *RCT* randomized control trial, *RF* radiofrequency, *RT* riboflavin-tryptophan, *SJW* St. John’s Wort, *W* week.

^a^ Reported as mean age in years ± standard deviation and/or age range

^b^ Light, PDT, and laser treatments are listed in order of application

^c^ Units for laser and light parameters: wavelength (nm), pulse duration (μs, ns, ms), energy (J, mJ), density (J\cm^2^, W/cm^2^, mW/cm^2^, spots/cm^2^), spot size (mm), repetition rate (Hz), duration of light exposure (min), light source distance from skin (cm).

^d^ Units for PDT parameters: photosensitizer concentration (%, mg/g) and incubation time (min, h).

^e^ Units for RF parameters: depth (mm), intensity/level (no units), duration (ms), density (MTZ/cm^2^, mJ/cm^2^), repetition rate (Hz)

^f^ Follow-up after final treatment session

^g^ FCL treatment began 1–2 months after last IPL treatment

^h^ Dual-mode consists of stamp-mode and moving-mode. Stamp-mode is the typical mode used with the 1450-nm diode laser.

**Table S2.** Results Summary for CSL

| Authors | Intervention(s) | *N* | Parameters/Dosages^a–d^ | Measurement Location | Measurement Method | CSL reduction (%)^e^ |
| --- | --- | --- | --- | --- | --- | --- |
| Light vs. Control | | | | | | |
| Ding et al. (2022) | Blue light vs. No treatment | 11 | - 415 ± 5 nm, 48 J/cm^2^, 40 mW/cm^2^, 20 min | Cheek^f^ | Collection 30-min after washing face with water and sitting in climate controlled conditions^g^ | - W4^h^: 60.9^†^ vs. (/) |
| Kwon et al. (2013) | Blue-red LED vs. Sham | 18 vs. 17 | - 420 nm, 0.91 J/cm^2^, 6.1 mW/cm^2^ (blue light); 660 nm, 1.22 J/cm^2^, 8.1 mW/cm^2^ (red light); 5 min | Cheek^f^ | Collection ≥ 5-h after washing face with soap;^i^ strip was placed for 30s using a constant pressure | - W8^h,^*: 12.5 vs. −4.2 - W12^h,^*: 19.9^†^ vs. −2.0 |
| Zane et al. (2008) | Red light vs. No treatment | 15 | - 600–750 nm, 20 J/cm^2^, 20 mW/cm^2^, 8 min, 40 cm | - Red light: forehead, chin - Control: upper back, presternal skin | – | - W16: 43.0^†^ vs. (/) |
| Light vs. Light | | | | | | |
| Wang et al. (2013) | 4 IPL + FCL (Group A) vs.  5 IPL + FCL (Group B)  vs.  6 IPL + FCL (Group C) | 5 vs. 6 vs. 4 | - IPL: 200–515 nm, 3–4 ms, 14–18 J/cm^2^ - FCL: 10,600 nm, 0.12–1.3 mm, 10–150 mJ, 300–400 Hz | Nasolabial fold | Ten consecutive measurements ≥ 30-min after washing face with neutral lotion and sitting in climate controlled conditions^g^ | - After IPL^h^: 19.6^†^ vs. 23.8^†^ vs. 16.0^†^ - 12 w FU^h^: 11.9^†^ vs. 16.1^†^ vs. 12.8 |
| PDT vs. Control | | | | | | |
| Kwon et al. (2016) | ALA-bu + daylight vs. VEH + daylight | 22 vs. 23 | - ALA-bu: 1.5% | Cheek^f^ | Collection ≥ 5-h after washing face with soap;^i^ strip was placed for 30s using a constant pressure | - W12^h,^*: 32.6^†^ vs. 7.3 |
| PDT vs. Light | | | | | | |
| Akaraphanth et al. (2007) | ALA + blue light vs. Blue light | 20 | - ALA: 10%, 1 h - Blue light: 409–419 nm, 48 J/cm^2^, 40 mW/cm^2^, 20 min, 10 cm | Forehead^j^ | Collection 15-min after washing face and sitting in climate controlled conditions^g^ | - W8: −4.1 vs. 3.9 - W12: 15.3 vs. 36.1 |
| Horfelt et al. (2009) | MAL + red light vs. Red light | 19 | - MAL: 160 mg/g, 3 h - Red light: 635 nm, 15 J/cm^2^, 63 mW/cm^2^ | Cheek^f^ | Subjects refrained from face washing or emollient use 12-h before measurement; strip was placed for 30s using a constant pressure | – |
| Na et al. (2011) | IAA + green light vs. Control gel + green light | 14 | - IAA: 0.015%, 15 min - Green light: 520 nm, 9 J/cm^2^, 15 min | – | – | - W6^h,^*: 34.1^†^ vs. 27.7 |
| Song et al. (2014) | Chlorophyll-*a* + blue-red LED vs. Blue-red LED | 24 | - Chlorophyll-*a*: 30 min - LED: 430 nm, 1170 J/cm^2^, 600 mW/cm^2^ (blue); 660 nm, 1080 J/cm^2^, 650 mW/cm^2^ (red); 30 min, 5 cm | Cheek^f^ | Strip was placed for 30s | - W6^h,^*: 37.4^†^ vs. 16.5^†^ |
| PDT vs. PDT | | | | | | |
| Jang et al. (2011) | ICG + 805-nm DL vs. IAA + Green light | 34 | - ICG: 0.2%, 15 min - IAA: 0.015%, 30 min - DL: 805 nm, 4 W/cm^2^, 10 mm - Green light: 520 nm, 35 mW/cm^2^, 30 min | Cheek^f^ | Subjects refrained from face washing 6-h before measurement | - W5: 42.3^†^ vs. 33.9^†^ - W8: 38.0^†^ vs. 33.3^†^ - W12: 36.8^†^ vs. 34.5^†^ - W16: 31.3^†^ vs. 30.4^†^ |
| Kim, Kim et al. (2023) | SJW + green-red LED vs. IAA + green-red LED | 31 | - SJW: 0.5%, 10 min - IAA: 0.015%, 10 min - LED: 630-nm (red) and 520-nm (green), 21 J/cm^2^, 35 mW⁄cm^2^, 10 min | Cheek^f^ | Subjects refrained from face washing 6-h before measurement | - W4: 27.9^†^ vs. 23.3^†^ - W8: 27.2^†^ vs. 26.9^†^ |
| Oh et al. (2009) | (Short incubation ALA + IPL vs. Long incubation ALA + IPL) vs. IPL | (9 vs. 11) | - ALA: 20%, 30 min (short), 3 h (long) - IPL: 590 nm cut-off filter, 30 ms, 12–15 J/cm^2^ | – | Subjects refrained from face washing 6-h before measurement | - W12: (35.5^†^ vs 45.7^†^) vs. 31.8^†^ - W16^h^: (36.5^†^ vs. 40.0^†^) vs. 28.9^†^ - W20: (31.5^†^ vs. 46.7^†^) vs. 37.5^†^ |
| Wangsuwan and Meephansan (2019) | RT + blue light vs. ALA + blue light | 34 | - RT: 0.1 mL sheet, 30 min - ALA: 13%, 30 min   Blue light: 415 nm, 48 J/cm^2^, 20 min, 5 cm | Cheek^f^ | – | - W5: 42.3^†^ vs. 41.4^†^ - W7: 54.4^†^ vs. 56.6^†^ |
| Laser vs. Control | | | | | | |
| Jung et al. (2012) | Dual-mode 1064-nm Nd:YAG laser + carbon suspension vs. No treatment | 20 | - Carbon suspension: 20 min - Laser: 1064 nm, 7 mm, 10 Hz; 300 μs, 1.8–2 J/cm^2^ (quasi-LP); 5 ns, 1.8–2.3 J/cm^2^ (QS) | Cheek^f^ | Collection ≥ 5-h after washing face with soap;^i^ Strip was placed for 30s using a constant pressure. | - W8^h,^*: 27.7^†^ vs. −3.8 |
| Kesty and Goldberg (2020) | 650 μs 1064-nm Nd:YAG laser vs. Sham | 10 vs. 10 | - 1064 nm, 650 μs, 11–64 J/cm^2^, 2–6 mm | – | – | - W12: 18.0 vs. 9.0 |
| Laser vs. Light | | | | | | |
| Kwon et al. (2019) | 1450-nm DL + blue light vs. Blue light | 24 | - Laser: 1450 nm, 280 ms, 5–7 J/cm^2^, 6 mm - Blue light: 450 nm, 0.6–1.2 J/cm^2^, 3.5–7 mW/cm^2^, 3 min | Cheek^f^ | Collection ≥ 5-h after washing face with soap;^i^ strip was placed for 30s using a constant pressure | - W9^h,^*: 45.7^†^ vs. 1.8 - W20^h,^*: 41.0^†^ vs. −11.4 |
| Laser vs. PDT | | | | | | |
| Chen et al. (2023) | QS 1064-nm Nd:YAG laser + ALA + red light vs. ALA + red light | 12 vs. 12 | - Laser: 1064 nm, 2 J/cm^2^, 7 mm - ALA: 2%, 40 min - Red light: 633 nm, 37 J/cm^2^, 105 mW/cm^2^, 6 min | Cheek^f^, forehead, nose | – | - W12*: 37.5 ± 49.1^†^ vs. 16.3 ± 42.6^†^ - W24: 18.3 ± 67.9^†^ vs. 17.4 ± 45.2^†^ |
| Laser vs. Laser | | | | | | |
| Kwon, Choi et al. (2018) | Dual-mode 1450-nm DL vs. Stamp-mode 1450-nm DL | 24 | - 1450 nm, 6 mm - Stamp mode: 280 ms, 5–7 J/cm^2^ and 14–15 J/cm^2^ - Moving mode: 65 ms, 45 W/cm^2^ | Cheek^f^ | Collection ≥ 6-h after washing face with soap;^i^ strip was placed for 30s using a constant pressure | - W9^h,^*: 40.3^†^ vs. 28.0^†^ - W20^h,^*: 47.8^†^ vs. 21.7^†^ |
| RF vs. Control | | | | | | |
| Choi et al. (2019) | NMRF vs. No treatment | 11 | 71 J/cm^2^, 6 MHz | Cheek^f^ | – | - W4*: 23.1 vs. 0.6 |
| RF vs. Laser | | | | | | |
| Kim, Lee et al. (2023) | FMR + FCL vs. FCL | 5 | - FMR: 1.6–2.0 mm, 4–6 - FCL: 10,600 nm, 100 mJ, 100 spots/cm^2^ (density) | Chin | – | - W20^*^: 12.5^†^ vs. −13.9 |
| Kwon, Park et al. (2018) | FMR vs. 1450-nm DL | 25 | - FMR: 0.8–2.0 mm, 20–50, 50–100 ms - Laser: 1450 nm, 250 ms, 7–12 J/cm^2^ | Cheek^f^ | Collection ≥ 5-h after washing face with soap;^i^ strip was placed for 30s using a constant pressure. | - W9^h^: 34.2^†^ vs. 42.3^†^ - W20^h,^*: 33.9^†^ vs. 22.1^†^ |
| Shin et al. (2012) | FMR vs. FCL | 20 | - FCL: 10,600 nm, 80 mJ, 100 spots/cm^2^ - FMR: 1.5–2.5 mm, 8, 25 MTZ/cm^2^ | – | – | - 12 w FU: 30.9^†^ vs. 31.0^†^ |
| RF vs. RF | | | | | | |
| Min et al. (2015) | FMR vs. BR | 20 | - FMR: 2–3, 50–70 ms - BR: 100 mJ/cm^2^, 100 Hz | Cheek^f^ | Collection ≥ 2-h after washing face with soap;^i^ strip was placed for 30s using a constant pressure | - W8^h,^*: 63.5^†^ vs. 34.2 - W12^h,^*: 65.4^†^ vs. 30.9 |

Abbreviations: *ALA* 5-aminolevulinic acid, *BR* bipolar radiofrequency, *DL* diode laser, *FCL* fractional carbon dioxide laser, *FMR* fractional microneedling radiofrequency, *FU* follow-up, *IAA* indole-3-acetic acid, *ICG* indocyanine green, *IPL* intense pulsed light, *LED* light-emitting diode, *MAL* Methyl-aminolevulinate, *N* number of participants who had sebum measured, *Nd:YAG* neodymium-doped yttrium aluminum garnet, *NMRF* non-ablative monopolar radiofrequency, *PDT* photodynamic therapy, *QS* Q-switch mode, *RF* radiofrequency, *RT* riboflavin-tryptophan, *SJW* St. John’s Wort, *VEH* vehicle cream, *W* week.

* *P* < 0.05 compared to other intervention

^†^ *P* < 0.05 compared to baseline

^a^ Light, PDT, and laser treatments are listed in order of application

^b^ Units for laser and light parameters: wavelength (nm), pulse duration (μs, ns, ms), energy (J, mJ), density (J\cm^2^, W/cm^2^, mW/cm^2^, spots/cm^2^), spot size (mm), repetition rate (Hz), duration of light exposure (min), light source distance from skin (cm).

^c^ Units for PDT parameters: photosensitizer concentration (%, mg/g) and incubation time (min, h).

^d^ Units for RF parameters: depth (mm), intensity/level (no units), duration (ms), density (MTZ/cm^2^, mJ/cm^2^), repetition rate (Hz)

^e^ Percent reduction from baseline at follow-up visits

^f^ The most prominent area of the zygoma

^g^ Climate control conditions were 20–21°C room temperature and 40–50% relative humidity.

^h^ Raw data extracted from WebPlotDigitizer

^i^ No additional face washing, cosmetics, or topical products allowed until after CSL measurement

^j^ Glabella area, 1 cm above the middle of the right and left eyebrows
